# Rural-Urban Disparities in ADRD Mortality by Nurse Practitioner and Physician Assistant Practice Policies

**DOI:** 10.1093/geroni/igaf122.025

**Published:** 2025-12-31

**Authors:** Nasim Ferdows

**Affiliations:** Northeastern University, Boston, Massachusetts, United States

## Abstract

Alzheimer’s Disease and Related Dementias (ADRD) mortality has risen over the past decade, with rural areas consistently experiencing higher rates than urban areas. Limited healthcare access, particularly in states with restrictive Nurse Practitioner (NP) and Physician Assistant (PA) policies, may contribute to these disparities. Using CDC WONDER data from 2010 to 2020, we analyzed ADRD mortality across rural and urban areas and examined the association between state-level NP and PA practice policies and mortality rates. NP practice authority was categorized as either Full Practice Authority (FPA), where NPs can practice and prescribe independently, or restricted, where physician oversight is required. PA practice was classified as restrictive, moderate, or permissive based on state regulations. ADRD mortality was consistently higher in rural areas, with rural mortality rates increasing from 95.2 per 100,000 in 2010 to 123.4 per 100,000 in 2020, compared to an increase from 76.5 to 98.7 per 100,000 in urban areas. States with restrictive NP and PA policies had the highest ADRD mortality rates, particularly in rural settings, while states granting FPA to NPs and permissive practice authority to PAs had lower mortality rates. In rural areas, states with FPA had an average ADRD mortality rate of 110.3 per 100,000, compared to 126.7 per 100,000 in restrictive states. These findings suggest that expanding NP and PA scope of practice may improve healthcare access and reduce ADRD mortality, particularly in rural communities, highlighting the need for policy changes to address rural-urban disparities.

